# Sub-sampling graph neural networks for genomic prediction of quantitative phenotypes

**DOI:** 10.1093/g3journal/jkae216

**Published:** 2024-09-09

**Authors:** Ragini Kihlman, Ilkka Launonen, Mikko J Sillanpää, Patrik Waldmann

**Affiliations:** Research Unit of Mathematical Sciences, University of Oulu, FI-90014 University of Oulu, Finland; Research Unit of Mathematical Sciences, University of Oulu, FI-90014 University of Oulu, Finland; Research Unit of Mathematical Sciences, University of Oulu, FI-90014 University of Oulu, Finland; Research Unit of Mathematical Sciences, University of Oulu, FI-90014 University of Oulu, Finland

**Keywords:** deep learning, graph convolution, quantitative genetics, predictive Breeding

## Abstract

In genomics, use of deep learning (DL) is rapidly growing and DL has successfully demonstrated its ability to uncover complex relationships in large biological and biomedical data sets. With the development of high-throughput sequencing techniques, genomic markers can now be allocated to large sections of a genome. By analyzing allele sharing between individuals, one may calculate realized genomic relationships from single-nucleotide polymorphisms (SNPs) data rather than relying on known pedigree relationships under polygenic model. The traditional approaches in genome-wide prediction (GWP) of quantitative phenotypes utilize genomic relationships in fixed global covariance modeling, possibly with some nonlinear kernel mapping (for example Gaussian processes). On the other hand, the DL approaches proposed so far for GWP fail to take into account the non-Euclidean graph structure of relationships between individuals over several generations. In this paper, we propose one global convolutional neural network (GCN) and one local sub-sampling architecture (GCN-RS) that are specifically designed to perform regression analysis based on genomic relationship information. A GCN is tailored to non-Euclidean spaces and consists of several layers of graph convolutions. The GCN-RS architecture is designed to further improve the GCN’s performance by sub-sampling the graph to reduce the dimensionality of the input data. Through these graph convolutional layers, the GCN maps input genomic markers to their quantitative phenotype values. The graphs are constructed using an iterative nearest neighbor approach. Comparisons show that the GCN-RS outperforms the popular Genomic Best Linear Unbiased Predictor method on one simulated and three real datasets from wheat, mice and pig with a predictive improvement of 4.4% to 49.4% in terms of test mean squared error. This indicates that GCN-RS is a promising tool for genomic predictions in plants and animals. Furthermore, GCN-RS is computationally efficient, making it a viable option for large-scale applications.

## Introduction

In quantitative genetics, pedigree-based associations have been used for many years to improve selection responses of phenotypes. It is crucial to know and understand the relationships within the populations to accurately predict polygenic breeding values and estimate genetic and environmental variance components. Usually, genetic relations are divided into additive genetic and dominance genetic components and assigned into matrices that correspond to individual members of the pedigree (Lynch and Walsh 1998).

In the early stages, pedigree-based quantitative genetics was focused on animal breeding but eventually found application in plant and tree breeding, human genetics, and evolutionary biology as well. Variance component-based quantitative genetics with known pedigree relationships has been an important part of the history of animal breeding, human genetics, evolutionary biology, and plant and tree breeding with some minor differences in each of these. Statistical inference during the last several decades has been dominated by linear mixed models, both in the frequentist restricted maximum likelihood (REML) and the Bayesian Gibbs sampling framework (Sorensen and Gianola 2002).

Genomic markers can now be scored for large parts of a genome thanks to high-throughput sequencing techniques. By using estimates of allele sharing between individuals, genomic markers, such as single-nucleotide polymorphisms (SNP), allow one to obtain empirical realized relationships rather than expected polygenic relationships. Genomics relationship coefficients are calculated in several ways, but most of them are based on standardized covariances, whose arrangements are called genomic relationship matrices (VanRaden 2008). Therefore, genotyping can provide direct estimates of relationship coefficients that can either replace or be combined with theoretical (expected) values derived from known pedigrees.

Meuwissen *et al.* (2001) introduced the concept of genome-wide prediction (GWP) and described how genomic markers can be used to predict quantitative phenotypes of individuals. Current genome-wide marker data include several thousand and occasionally millions of SNPs, and they are analyzed for numerous individuals whose numbers range from some hundreds to a few thousands (de Los Campos *et al.* 2013). Machine learning is being used successfully in several forms of genomic sequence analysis, including GWP, genome-wide association studies (GWAS), and other forms of automated high-dimensional parameter learning (Okser *et al.* 2014; Libbrecht and Noble 2015; Momen *et al.* 2018). In machine learning, deep artificial neural networks are among the most flexible methods and have become state of the art because of their outstanding prediction capabilities (LeCun *et al.* 2015). Their use in genomics is rapidly increasing (Eraslan *et al.* 2019; Yue *et al.* 2023).

Montesinos-López *et al.* (2021) compared deep learning (DL) methods, including Multilayer Perceptron (MLP), Convolutional Neural Network (CNN) and local CNN (LCNN) architectures, to conventional GWP models exemplified by Genomic Best Linear Unbiased Predictor (GBLUP), BayesA, and Extended GBLUP. They found that DL models better capture nonadditive effects and complex higher order relationships without additional interaction terms. A DL model copes well with large datasets and it can also efficiently incorporate external data and images without cumbersome preprocessing or feature engineering. This makes DL models better suited to integrating large quantities of omics data into the same model.

DL enables easy training of continuous, binary, categorical, and count models. A wide range of activation functions and hidden layers enable the use of DL networks to model with high precision. To model latent variables and patterns in data, a variety of optimizers as well as inputs are available. An SNP linkage imbalance can be captured with CNN topologies when it comes to DL models. By pooling operations without estimating other parameters, CNN topologies allow parameter sharing and compression, thereby reducing parameter estimates. Although MLP and CNN architectures have been successful in GWP (Montesinos-López *et al.* 2021), they rely mainly on grid or independent input data that do not take into account the pedigree structure of individuals. Genome-wide sequences are therefore not optimally leveraged for topological properties.

Graph Neural Networks (GNNs) have emerged as powerful DL tools that process network structures and network data. Their convolutional architecture inherits all the favorable properties of CNNs, while they are also able to exploit the graph structure of data. Recently, their use has increased dramatically and they seem to outperform existing methods in many areas. Several comprehensive reviews about the effectiveness of GNNs along with discussing the subfield of geometric DL have been produced (Wu *et al.* 2020; Chami *et al.* 2022).

In this paper, we propose two regression-based GNN architectures for GWP of individual phenotypes. The GNN maps input genomic markers to their phenotype values through GCN layers. The graphs utilize the realized empirical genomic relationships among various individuals, thereby mimicking pedigree-based quantitative phenotype prediction. We describe the process of creating the GNNs using a neighborhood graph that locates a predefined number of relatives closest in distance to an individual (vertex in graph terminology). Then, we show how to transform the GNNs to GCNs via embeddings. Further on, we outline how to train one global GCN model and one local sub-sampling GCN-RS model that aggregate messages from the individuals nearest the neighbors in the graph and tune the hyper-parameters using out-of-sample validation data. Finally, we evaluate the prediction and computing performance of the GCN models and two competing state-of-the-art methods on one simulated and three real datasets.

## Methods

### Graphical representation of data

The most common input for DL models has been either independent variables or Euclidean grid-like arrays of data. CNNs are data mining techniques that use convolutions within kernels to extract latent information. With the use of hidden convolution and pooling layers, spatially localized features can be identified. The inputs to classical CNNs often follow grid-like structures, for example in tasks such as image classification, image recognition, and object detection (Girshick *et al.* 2014). CNNs are the most popular method to solve a wide range of problems in these areas because of their capacity to provide accurate prediction results (Sharma *et al.* 2020). However, many types of data are structured following a more complex non-Euclidean pattern, whose expression in a format compatible with standard Euclidean-based DL is nonintuitive.

### Graphs

Graphs are mathematical tools that simplify the process of structuring, analyzing, and visualizing data (Anscombe 1973). A graph can be used to make predictions based on four types of information: vertices, edges, context, and connectivity. An ordinary CNN cannot take advantage of the topology and shape of the graph because graphs have no spatial locality since their spatial size and topology are arbitrary (Boccaletti *et al.* 2006). Non-Euclidean data that is arbitrarily structured is therefore difficult for traditional machine learning and DL models to learn from. More challenges arise from describing a graph’s connectivity, which usually results in a sparse adjacency matrix that is undesirably space-inefficient. In particular, there is no specific order in which vertices appear in graphs. As far as deep neural networks are concerned, these different matrices are not permutation-invariant, that is, they do not yield the same result (Bronstein *et al.* 2017).

The process of presenting data in a graphical format involves identifying the entities (vertices in the graph), identifying the relations (edges in the graph), and creating the actual graph. Let G=(V,E) be a graph where $V=\{v_{1},v_{2},\ldots ,v_{N}\}$ is a set of vertices and E is a set of (undirected) edges with elements $e_{ij}=\{v_{i},v_{j}\}\in E$ implying that the vertices $v_{i}$ and $v_{j}$ are connected. One can store this connection information in an adjacency matrix *A*: if two vertices $v_{i}$ and $v_{j}$ are connected, the corresponding element $A_{ij}=1$, otherwise $A_{ij}=0$.

### Nearest Neighbor graphs

Nearest Neighbor graphs have vertex points with associated observations. Each vertex has a directed edge extending between it and *K* of its Nearest Neighbors. Neighbor methods predict a label based on a predefined number of training samples closest to the new point. Users can define the number of samples (*K*-Nearest Neighbor learning) or use the local density of points (radius-based neighbor learning). Generally, any metric measure can be used, but the Euclidean distance is the most common choice. Let the dataset with *N* instances be denoted by matrix $X=[x_{1},\ldots x_{N}]$. A Nearest Neighbor graph *G* is constructed using *X*. Each vertex $v_{i}$ in *G* is represented by an instance vector $x_{i}$ in *X*. As a first step, no edge connects any two vertices in *G*. The next step is to search for *K* nearest neighbors for each instance. A connection is established between the instance and its *K* nearest neighboring instances by placing edges between them in the graph *G*. A weight matrix *U*, where the entries indicate how close two neighboring instances are, can also be calculated. Individuals are represented as vertices, while genetic relationships between them are represented as edges, preserving their original structure and properties.

### Graph neural networks

A GNN is a type of machine learning model that can be optimized for all attributes of a graph (vertices, edges and global context) while still maintaining the graph symmetries (Micheli 2009). In this type of neural network model, a graph is taken as input together with possible information ascribed to its vertices, edges and global constituents. The development of GNNs was recently further extended in order to deal with local patterns as well as hierarchies of patterns within graphs. As graphs are locally connected structures, it has been demonstrated that graph representations (or embeddings) require convolutions to learn local patterns regardless of changes in size or position of the input. This phenomenon is known as translation invariance, which led to the use of stacked convolutions for interpretation and learning of graphical information (Kipf and Welling 2016).

### Graph convolutional networks

Graph convolutional networks (GCNs) are considered one of the most basic variants of GNNs. GCNs have recently been proposed as graph models for semi-supervised learning (Kipf and Welling 2016). A GCN exploits the structural information of a graph by applying a convolution over it. Instead of working with a fixed 2D array, GCN utilizes graphs to enhance its prediction abilities by taking into account both a vector and its neighbors.

In terms of weight sharing, GCNs are similar to CNNs (Duvenaud *et al.* 2015). The procedure consists of multiplying the input neurons with a set of weights via a convolution operator which is called a filter or a kernel. In GNNs, filters serve as sliding windows across the entire feature vector, allowing neighboring cells to provide features. Weight sharing refers to the use of the same filter throughout a layer. GCNs are a generalized version of GNN that can work on data with nonregular graph structures, whereas CNNs work on data with regular grid structures.

Traditional NNs are trained using individual data features and labels. The layer propagation rule for each layer of a traditional MLP can be summarized as


(1) $$z^{\left(l+1\right)}=\sigma (W^{\left(l\right)}z^{\left(l\right)}),$$

where $z^{\left(l+1\right)}$ is the output of the layer with index l+1, $z^{\left(0\right)}=x_{i}$ is a vector of length *P* with input data, $W^{\left(l\right)}$ is a weight matrix, and *σ* is a nonlinear activation function. The final hidden layer is linearly connected to the output $y_{i}$.

However, in a GCN, training is based not only on the features of an individual $v_{i}$, but also on the features of the neighboring vertices $v_{\;j,\ldots ,K}\underset{\neq i}{\subset }\;V$ where *K* denotes the number of neighbors. Such a framework is designed to learn from a set of features on a graph G=(V,E) and produce a vertex-level output *Z* (an N×F feature matrix, where *F* is the number of output features per vertex). A GCN receives as input a feature matrix $X^{\left(i\right)}$ for every vertex $v_{i}$, with dimensions N×P where *N* is the number of vertices and *P* is the number of input features, along with a matrix representing the graph structure, typically an adjacency matrix *A*. The sparseness of *A* means that the actual input size of $X^{\left(i\right)}$ is K×P. From a quantitative genetics perspective, this means that we can utilize the information from both a set of restricted genomic relationships via the adjacency matrix and the raw marker data via the feature matrix.

Due to GCNs’ similarity to CNNs, this layer-wise propagation rule can be written as


(2) $$H^{\left(l+1\right)}=\sigma (\tilde{A}H^{\left(l\right)}W^{\left(l\right)}),$$

where $\tilde{A}=A+I$, *I* is the identity matrix, $H^{\left(l+1\right)}$ is the output of the layer with index l+1 and $H^{\left(0\right)}=X$.

For each hidden layer, NN computes its output as $Z^{\left(l+1\right)}=\sigma (W^{\left(l\right)}Z^{\left(l\right)})$ while GCN computes it as $H^{\left(l+1\right)}=\sigma (\tilde{A}H^{\left(l\right)},W^{\left(l\right)})$

When generalized, this results in the forward propagation rule, introduced by Kipf and Welling (2016) as


(3) $$H^{\left(l+1\right)}=\sigma \left({\tilde{D}}^{-\frac{1}{2}}\tilde{A}{\tilde{D}}^{-\frac{1}{2}}H^{\left(l\right)}W^{\left(l\right)}\right),$$

where $\tilde{D}$ is the diagonal vertex degree matrix of $\tilde{A}$. Due to this operation, *A* becomes normalized.

### Embeddings for a GCN

Finding an efficient way of encoding graph structures that can be exploited by machine learning algorithms is the primary challenge in analyzing graph-based data. Specialized approaches are often needed to extract relevant information from graphs. A graph embedding is a type of vector representation of a graph that translates high-dimensional vectors into relatively low-dimensional spaces by using graph data as input. The GCN generates a vector of numerical values that represent the graph’s vertices and relationships. The message-passing operation is repeated in GCNs with more than one layer, aggregating the values of neighbors of neighbors. Finally, the output layer generates an embedding of the vector, which is the vector representation of the data and other information.

Graph embedding is an effective way to encode vertices in a way that approximates similarity with the original network. Vector space relationships must map to original network relationships via a similarity function. Embeddings can be obtained in a variety of ways, each with a varying level of granularity (e.g. Node2vec Grover and Leskovec 2016 and Graph2vec Narayanan *et al.* 2017). The GCN can do this by aggregating all its neighbors at a vector and propagating it across all its layers through forward message passing (Bronstein *et al.* 2017).

In this paper, we explore the use of the Nearest Neighbor algorithm to construct the graph and establish the relationship between individuals (vertices) based on how close they are to each other. Thus, equation 2 can be expressed in terms of GCN embeddings as


(4) $$h_{v}^{\left(1\right)}=\sigma \left({\text{W}}^{\left(1\right)}\sum_{u\in ℵ\left(\upsilon \right)}\frac{h_{\upsilon }^{\left(l-1\right)}}{\left\|ℵ\left(\upsilon \right)\right\|}+B^{\left(l\right)}h_{\upsilon }^{\left(l-1\right)}\right)\forall l>0$$

where $h_{v}^{\left(l\right)}$ is the *l*th layer embedding of vertex *v*, N(v) is the neighborhood of *v*, $\sum_{u\in N(v)}\frac{h_{u}^{\left(l-1\right)}}{\left\|N(v)\right\|}$ is the average of the previous layer embedding of the neighbors, $h_{v}^{\left(l-1\right)}$ is the previous layer embedding of vertex *v*, and $W^{\left(l\right)}$ and $B^{\left(l\right)}$ are weight matrices. Each vector’s embeddings are obtained after l layers of neighborhood aggregation. Using these embeddings, one can then train the aggregation parameters using stochastic gradient descent.

### Graph sampling

It is imperative to note that the original GCN (Kipf and Welling 2016) is inherently transductive. This means that the embeddings can only be generated for the vertices that are present in the current fixed graph during training. If the graph evolves in the future the entire graph will need to be retrained. Training is ineffective in the whole-graph GCN since it uses a graph convolution process to learn the embeddings of nodes at each layer from top to bottom. As a result, the top-down approach of computing embeddings is slow and can lead to computational bottlenecks. It also makes it difficult for the model to capture the global context of the graph, as only the information from the neighboring nodes are used to compute the embeddings. Research has been conducted to address the limitations of the traditional GCN model to extract features from graphs. Inductive learning provides a better alternative to generalize predictions beyond a fixed graph and it has been used in GraphSage (Hamilton *et al.* 2017). Here, vertices are represented as aggregates of their neighborhoods and the embeddings are


(5) $$h_{v}^{\left(l\right)}=\sigma \left(W^{\left(l\right)}\cdot \left[\underset{u\in N(v)}{\text{AGG}}\;\{h_{u}^{\left(l-1\right)}\},h_{v}^{\left(l-1\right)}\right]\right)\;\forall \;l>0,$$

where $\underset{u\in N(v)}{\text{AGG}}\;\{h_{u}^{\left(l-1\right)}\}$ is an aggregation of *v*’s neighbor’s embeddings at the prior layer. There are several options for aggregation function, but the mean or maximum are most convenient. As a result, even if a new data vector appears in the graph after training, its neighbors can still properly represent it. The key is to concatenate the data vectors of the neighbors of each vertex with its own data vector, rather than passing both into the same aggregating function. Furthermore, GraphSage performs neighborhood sampling, making its algorithm capable of scaling up to billions of vertices. In other words, the model is trained on all vertices of the graph, but the sampling provides subsets of neighbor information. Hence, it is easy to share parameters across computational graphs or architectures. When a new graph architecture is introduced, predictions can be generated using borrowed parameters. In summary, the primary difference is that GraphSAGE is sampling-based and uses a simple mean of nearby embeddings, whereas the traditional GCN is fixed and uses a weighted mean based on the normalization coefficients (Chen *et al.* 2018).

### GCN architecture

In our work, we have developed two different models. Firstly, a traditional whole-graph GCN model is deployed over each genomic dataset in order to analyze how well it predicts the phenotype of individuals. In the second model, batches of individuals with marker data are selected randomly and the resulting sub-graphs are fed into the GCN using a random sampling method. The final model prediction is the value that is averaged over the best models across all batches.

The following steps are used to build the GCN regression models

- **Train, validation, and test splitting.** First, we randomly divide the data (V,X) and the labels *Y* into training, validation, and test sets $\left(V_{\left(tr\right)},X_{\left(tr\right)},Y_{\left(tr\right)}\right)$, $\left(V_{\left(va\right)},X_{\left(va\right)},Y_{\left(va\right)}\right)$ and $\left(V_{\left(te\right)},X_{\left(te\right)},Y_{\left(te\right)}\right)$, respectively. One hundred individuals were first allocated to be test data in all our four different datasets. The remainders were then divided into 80 % training and 20 % validation since this form of partition is easier to handle in PyTorch. Supplementary Table 1 displays details on the vertex, edge, and degree of the full data sets and Supplementary Figs. 1, 2, 3, 4 illustrate the graphical structure of the respective datasets for the first 100 individuals.
- **Graph construction for feature extraction.** Using the Nearest Neighbor algorithm, we construct the graphs *G* based on the Euclidean distance between the individuals using genomic marker data. The best number of neighbors was tuned. The adjacency matrix can be generated as usual by
  (6) $$A_{ij}=\left\{ \begin{array}{cc} 1 & \text{if vertices}\;i\;\text{and}\;j\;\text{are connected in graph}\;G,\\ 0 & \text{otherwise}. \end{array}\right.$$
  Each vector represents an individual object and each edge represents a discretized relationship between individuals within a neighborhood. This process extracts initial vector features and marker data information. The adjacency matrix of the individuals is then summed with the identity matrix as $\tilde{A}=A+I$. Then, a graph traversing algorithm sets overlapping edges between the datasets to zero to avoid overfitting. We use the Nearest Neighbor algorithm to create the graph and the NetworkX library to visualize and manipulate it. Details on the vertex, edge, and degree of the full datasets and illustration of the graphical structure of the respective datasets for the first 100 individuals in the Supplementary material.
- **GCN structure.** We use two graph convolutional layers in our model to specify graph features *X* and adjacency matrix $\tilde{A}$. Our Nearest Neighbor graph is used to propagate messages and aggregate feature information of neighbors. In order to model complex dependencies between vertices, the neighbors of a vector are progressively aggregated to estimate the hidden state of vertices. As GCN transmits information to neighboring vertices and updates each vector based on the adjacency matrix (Liao *et al.* 2019), geometrical dependencies among objects can be effectively captured. Our output of the GCN is then passed through a rectified linear units (ReLU) activation layer and a dropout layer. A dropout regularization renders a neuron inactive by stochastically multiplying its input by 0 (Baldi and Sadowski 2013). ReLU activation is a piecewise linear function that will either output the input directly or set negative inputs to 0 (Hahnloser *et al.* 2000). The output of the GCN is then converted into a 1D vector by passing it through a linear layer. For predictive modeling, we have used regression since the target value of the phenotype predictions is continuous. Using the Mean Squared Error (MSE) loss, we minimize the discrepancy between predictions made by the model under training and the true phenotype values.

### Model training

GCN models are trained iteratively. Each backpropagation iteration of a model includes a forward and backward pass. Training leads to a reduction of the MSE loss. In order to reduce overfitting of the model, we use the adaptive moment estimation optimizer (ADAM) (Kingma and Ba 2014), which adapts decay of the learning rate using moving average and bias correction. Our training procedure also implements early stopping before the model starts overfitting. During our study, we found that the GCN approach is very efficient at learning graph representations. However, it involves nontrivial computational and storage costs. Additionally, it causes gradient descent to take a longer time to converge since parameters are only updated once in each iteration.

We also noticed that all intermediate embeddings of the entire graph are used to compute the gradient, making it difficult to scale to large graphs. Hence we developed a random sampling method (GCN-RS) to overcome these limitations. Here, the training process is divided into two phases, namely local aggregation and global aggregation. Through the random sampling phase, *N* sub-graphs are created and partial vertices of an entire graph are selected. In the local aggregation phase, each sub-graph’s training set is fed into two convolutional layers which recursively aggregate the neighboring features. A vector’s embedding in the second last layer of the GCN is updated by combining neighborhood features generated as the result of the aggregation with the vector’s previous embedding. In the global aggregation phase, the embeddings of *N* GCN models are loaded and passed through a linear aggregation layer as the activation function. This is the phase of the process where the mean of all the output embeddings of each model is calculated, and the final MSE is then calculated based on the overall mean. Algorithm 1 describes the traditional GCN model and Algorithm 2 shows in detail the steps in the random sampling sub-graph GCN-RS algorithm.

**Algorithm 1:** GCN - using the entire dataset

Given: Data (V,X); Labels *Y*;
- Output: GCN with trained weights and test MSE
- Pre-Processing: Build overall NN graph G(V,E,X) with neighbors =K
- Partition vertices, their genomic data and phenotype labels randomly into $\left(V_{\left(tr,va,te\right)},X_{\left(tr,va,te\right)}),Y_{\left(tr,va,te\right)}\right)$
- Use a graph traversing algorithm to set edges $E_{\left(tr\right)}$ between $V_{\left(tr\right)}$ and $V_{\left(va,te\right)}$, and between $V_{\left(va\right)}$ and $V_{\left(te\right)}$ to zero
- Remove $Y_{\left(va,te\right)}$ from graph
**for** each epoch **do**
- Train GCN on $G(V,E_{\left(tr\right)},X)$ with $Y_{\left(tr\right)}$ using the ADAM optimizer
- Use predicted ${\hat{Y}}_{\left(va\right)}$ and $Y_{\left(va\right)}$ to calculate validation MSE
- Tune hyper-parameters: learning rate *γ*, epoch iterations *it* and number of neighbors *K* based on validation MSE
- Use GCN with optimized hyper-parameters and predict ${\hat{Y}}_{te}$
- Calculate test MSE from ${\hat{Y}}_{\left(te\right)}$ and $Y_{\left(te\right)}$

**Algorithm 2:** GCN-RS - using random sampling of the dataset

- Given: Data (V,X); Labels *Y*;
- Output: Sub-GCN models with trained weights and test MSE
- Pre-Processing: Build overall NN graph G(V,E,X) with neighbors =K
- Preprocessing: Split *G* into train $G_{\left(tr\right)}$, validation $G_{\left(va\right)}$ and test $G_{\left(te\right)}$ using vertex splitting algorithm
**for** each sub-graph **do**
- $G_{\left(sg\right)}(V_{\left(sg\right)},E_{\left(sg\right)})$← Sample sub-graph of $G_{\left(tr\right)}$ using the vertex splitting algorithm **for** each epoch **do**
- Train sub-GCN on $G_{\left(sg\right)}$ and $Y_{\left(sg\right)}$ with the ADAM optimizer
- Use predicted ${\hat{Y}}_{\left(va\right)}$ and $Y_{\left(va\right)}$ to calculate validation MSE
- Tune hyper-parameters: learning rate *γ*, epoch iterations *it* and number of neighbors *K* based on validation MSE for each sub-graph
- Use each sub-GCN with optimized hyper-parameters and predict an ensemble of ${\hat{Y}}_{\left(sg,te\right)}$
- Take the average over the ensembles ${\hat{Y}}_{\left(sg,te\right)}$ and calculate test MSE using $Y_{\left(te\right)}$

### Hyper-parameter tuning

The software framework Optuna (Akiba *et al.* 2019) is used to automate the process of selecting suitable values for hyper-parameters. Optuna allows users to optimize for the best hyper-parameter settings while pruning unproductive trials. Compared to GridSearch, this speeds up optimization time and performance. Moreover, optimization histories can be plotted for a better understanding. Optuna uses Bayesian optimization (BO), where hyper-parameter values are iteratively mapped to the objective function. It forms a posterior distribution over the objective function based on beliefs about the function’s behavior. An acquisition function is then constructed using the posterior distribution to determine the best improvement point. By focusing on areas where the hyper-parameters give better results, the BO algorithm select the best parameter combinations. An early stopping method is used for pruning purposes.

In the current study, the objective function to be minimized is the test MSE loss. The hyper-parameters that we tuned for this study are:

- Learning rate—Determines the number of learning iterations required in the training phase to achieve minimum training MSE.
- Number of epochs—Each epoch is the number of times the neural network model passes the whole training dataset through iteration. Individual sub-graphs are passed through each epoch for the GCN-RS model and the whole training dataset is passed forward and backward through the neural network once (i.e. one epoch).
- Number of neighbors—In order to create a nearest neighborhood graph, it is necessary to determine the optimal number of neighbors.
- Number of sub-graphs (minibatches)—For the GCN-RS model, passing different sizes of the sub-graphs to be trained influences the network’s learning capacity.

## Evaluation

### BGBLUP and BRKHS

The GCN models were evaluated by comparing their performance with the results of two current state-of-the-art methods. The first method is Bayesian Genomic Best Linear Unbiased Prediction (BGBLUP) estimated using the Integrated Nested Laplace Approximation (INLA) (Mathew *et al.* 2015). INLA has established itself as an alternative to other methods such as Markov chain Monte Carlo because of its speed and ease of use via the R-INLA package. Although the INLA methodology focuses on models that can be expressed as latent Gaussian Markov random fields (GMRF), this encompasses a large family of models that are used in practice. With the R-INLA, Bayesian models were fitted with the genomic relationship matrix as a structured random effect. As default settings, an uninformative Log Gamma (1,5e-05) prior was chosen for the log precision (i.e. inverse variance) and a Normal (0,10) prior was chosen for the mean. We also implemented a Bayesian Reproducing Kernel Hilbert Space (BRKHS) regression using the BGLR package in R (Pérez and de los Campos 2014). This model is equivalent to a Gaussian process model and can be interpreted as a nonlinear BGBLUP (Wang and Jing 2022). We used the default settings in BGLR and ran the MCMC chains for 20,000 iterations where the first 5,000 iterations were discarded as burn-in. The dataset was randomly partitioned into 80% training and 20% test and scenarios were repeated 10 times for both BGBLUP and BRKHS. The results are presented as the mean over the 10 repetitions.

### Sparse Convolutional Neural Networks for Genome-Wide Prediction

The Sparse Convolutional Neural Networks for Genome-Wide Prediction (CNNGWP) implements deep learning techniques, and it uses Bayesian optimization to optimize the hyper-parameters of the neural networks (Waldmann *et al.* 2020). Ensemble predictions that are based on model averages further reduce prediction error and improve prediction accuracy. We have implemented this deep learning model using PyTorch (Paszke *et al.* 2019) and tuned the hyper-parameters using the bayes_opt python library (Nogueira 2014). The datasets for CNNGWP were partitioned into train, validation and test following the GCN procedure.

### Evaluation metrics

Using three different metrics, we compared the proposed method with state-of-the-art methods to determine if it is more efficient than existing approaches. The results also indicate that the proposed method can achieve greater accuracy while maintaining optimal computational efficiency.

We use the MSE to evaluate the model’s performance because it is the most common loss function in regression studies. It allows us to determine how well the predicted values match the actual values. Pearson’s correlation coefficient is a standardized measure that complements the MSE. A high correlation coefficient indicates a high prediction accuracy and it can be directly compared between different traits. However, this metric is strictly linear and can be misleading if the underlying data follows a nonlinear relationship or has a high variance between predicted observations (Waldmann 2019).

We have also used distance correlation implemented in the dcor() function in R package energy (Székely *et al.* 2007). Distance correlation is a measure of the strength of the relationship between two variables when taking into account the distance between them. Using this metric, one can identify outliers and spurious correlations. It is also possible to use it for comparison of different models without assuming a linear relationship between predicted and true observations.

## Materials

### QTLMAS dataset

In conjunction with the XVIth QTL-MAS workshop, a common dataset was simulated and made publicly available (Usai *et al.* 2014). Four generations, consisting of 20 males and 1000 females, were generated by mating each male with 50 females. The genome consisted of 5 chromosomes, each of 100 Mb size and carrying 2,000 equally distributed SNPs. Three traits were simulated in order to mimic milk yield, fat yield and fat content. Genetic effects were generated from 50 QTLs with some pleiotropic effects that influence multiple traits. In this study, we used fat yield (Tr2) as our quantitative phenotype, which has a simulated heritability of 0.35.

### Wheat dataset

This dataset consists of phenotypic, genotypic, and pedigree information for 599 wheat lines from the CIMMYT wheat breeding program. The dataset was made publicly available by (Crossa *et al.* 2010). Quantitative phenotypes were centered and normalized to unit variance within the environment as lines were evaluated for grain yield in four different environments. We averaged the quantitative phenotypic values over the four different environments to reduce the error variance and used that value as our quantitative trait.

### Mice dataset

This dataset can be found from BGLR package (Pérez and de los Campos 2014). The dataset consists of genotypes and phenotypes from 1,814 mice. Each mouse was genotyped at 10,346 SNPs and SNPs with minor allele frequency (MAF) below 0.05 were removed. We decided to use body length (BL) as quantitative phenotype, which was precorrected for significant fixed effects (month, year, gender, coat color, cage density, and litter size) that resulted in a heritability of 0.35.

### Pig dataset

The pig dataset contains 3,534 individuals with high-density genotypes (PorcineSNP60 chip), phenotypes of five anonymized traits and a pedigree derived from a single nucleus line including their parents and grandparents (Cleveland *et al.* 2012). Missing SNPs were imputed based on their probability scores. The markers were anonymized by randomizing the map order and recoding the SNP identities. SNPs were further reduced in our study using a more stringent MAF of 0.01, resulting in a final set of 50,281 SNPs. For our study, we chose the fourth trait (Tr4) as our quantitative trait with a heritability of 0.58. These phenotype values were first precorrected for environmental factors and rescaled before neural network analysis. A total of 3152 individuals were obtained after discarding those with missing phenotype data.

## Results

By performing some preliminary tests, we found that 0.0025 is the ideal learning rate for obtaining the minimum loss. The optimal number of epochs was 250. In order to prevent information loss, we found that three neighbors are the best number for graph creation. A batch size of 10 was found to be most effective for sub-graph sampling. This tuning improves model performance effectively. Following the hyper-parameter tuning, all models show lower test MSE values, indicating more accurate predictions and less overfitting. The two methods proposed in this paper aim to achieve a tradeoff between training time and accuracy. The outcome is highly dependent on the data size, level of errors and computational resources.

### QTLMAS dataset

The best result was obtained for GCN-RS based on test MSE (74.667) compared to the BGBLUP (78.121), CNNGWP (76.541), and GCN (81.061) methods (Table 1). The improvement in terms of percentage between GCN-RS and BGBLUP is only 4.4%. The Pearson and distance correlations are considerably higher for GCN-RS (0.431, 0.411) in comparison with the CNNGWP (0.416, 0.403), BGBLUP (0.326, 0.306), and GCN (0.381, 0.321) methods (Tables 2 and 3). The GNN-RS model is able to better capture the complex interactions between genetic markers and phenotypes due to its use of the random sampling approach. The use of random sampling enables the model to learn long-term temporal dependencies in the data, thereby allowing it to better capture the complex interactions between genetic markers and phenotypes. These results are supported both in terms of accuracy and the minimum test loss.

**Table 1.** MSE calculated between predicted and the original values of the test set individuals for different datasets (Mice, QTLMAS, Wheat, and Pig) for competing methods.

Dataset	MSE				
	BGBLUP	BRKHS	CNNGWP	GCN	GCN-RS
QTLMAS	78.121	76.601	76.541	81.061	**74.667**
Wheat	0.328	0.295	0.177	0.214	**0.166**
Mice	0.233	0.236	0.208	0.208	**0.194**
Pig	4.296	4.386	3.345	3.754	**3.236**

The best performances are indicated in bold.

**Table 2.** Pearson correlation calculated between predicted and the original values of the test set individuals for different datasets (Mice, QTLMAS, Wheat and Pig) for competing methods.

Dataset	Pearson correlation				
	BGBLUP	BRKHS	CNNGWP	GCN	GCN-RS
QTLMAS	0.326	0.396	0.416	0.381	**0.431**
Wheat	0.397	**0.477**	0.431	0.398	0.456
Mice	0.290	0.293	0.358	0.258	**0.384**
Pig	0.459	0.456	0.461	0.427	**0.497**

The best performances are indicated in bold.

**Table 3.** Distance correlation calculated between predicted and the original values of the test set individuals for different datasets (Mice, QTLMAS, Wheat and Pig) for competing methods.

Dataset	Distance correlation				
	BGBLUP	BRKHS	CNNGWP	GCN	GCN-RS
QTLMAS	0.306	0.365	0.403	0.321	**0.411**
Wheat	0.447	0.435	0.471	0.428	**0.496**
Mice	0.273	0.273	0.378	0.298	**0.404**
Pig	0.432	0.432	0.439	0.405	**0.447**

The best performances are indicated in bold.

### Wheat dataset

GCN-RS also yielded the smallest test MSE (0.166) for the wheat data, while the test MSE was 0.177, 0.214, and 0.328 for the CNNGWP, GCN, and BGBLUP, respectively (Table 1). This means that the GNN-RS shows that there is almost a 50% improvement against the BGBLUP method and a more than 20% improvement when compared to the GCN method (Supplementary Fig. 5). The distance correlation coefficient was also highest for GCN-RS (0.496). However, the Pearson correlation coefficient was highest for BRKHS (Tables 2 and 3). It has been observed before that the Pearson correlation coefficient can provide misleading results for model selection due to the bias-variance tradeoff in statistical learning (Waldmann 2019).

### Mice dataset

Once again, the test MSE comparison between the methods shows that the GCN-RS (0.194) is best (Table 1), which from a percent perspective leads to an improvement of 17% (Supplementary Fig. 5). It can also be noticed that the overall GCN (0.208) does provide a viable alternative to the CNNGWP (0.208), which in comparison to the other results can be considered as the best nongraphical model. The BGBLUP (0.233) and BRKHS (0.236) perform worst in terms of test MSE. The resulting correlation coefficients are the highest for GCN-RS (0.384 and 0.404, respectively) (Tables 2 and 3), a result that is in line with the QTLMAS data.

### Pig dataset

The results for the test MSE for this dataset also shows that GCN-RS (3.236) performs best. CNNGWP (3.345) is second best. GCN (3.754) is slightly worse but also considerably better than BGBLUP (4.296) and BRKHS (4.386) (Table 1). The percentage comparison between the methods shows an intermediate differences of 25% improvement between GCN-RS and BGBLUP (Supplementary Fig. 5). The Pearson and distance correlations where GCN-RS (0.497 and 0.447, respectively) which are higher than BGBLUP (0.459, 0.432), BRKHS (0.456, 0.432), CNNGWP (0.461, 0.439), and GCN (0.427, 0.405) (Tables 2 and 3). The pig dataset is larger than the other datasets. This means that the increased number of training batches facilitate training of the GCN-RS. However, it also means that the current implementation of GCN model will not be able to make use of the data beyond these sizes, which may limit its scalability and effectiveness.

### Train/test split proportions

Table 4 shows the effect of different proportions of train/test splits. Having a larger training set leads to higher model efficiency. The differences are not huge, but it can be seen that it is best to focus on a large training splits. These results indicate that the model’s performance does not drastically decline if it has a smaller training set. Due to the Wheat data being smaller than the other datasets, there is a greater variability in predictions when the training size decreases of these data. However, datasets of this size are difficult to fit reliably with any method and the results should be interpreted in light of this.

**Table 4.** Comparing the effect of having different proportions of the training/test sizes for different datasets (Mice, QTLMAS, Wheat and Pig) for competing methods.

Dataset	Different proportions of training/testing sizes				
	90/10	80/20	70/30	60/40	50/50
QTLMAS	73.404	74.667	75.503	75.812	76.986
Wheat	0.163	0.166	0.181	0.226	0.250
Mice	0.183	0.194	0.205	0.206	0.222
Pig	3.219	3.234	3.337	3.502	3.718

## Discussion

Two different approaches were used to create the GCN models in this study (Supplementary Fig. 6). In the first case, the entire dataset was passed to a GCN model for training, and in the second case, the training dataset was randomly split into 10 units that formed 10 different GCN-RS models that worked as ensembles for a final averaged prediction estimate. We found that the GCN model, where the entire input was passed, performs poorly compared to all the other methods. The reason for this is probably that a graph convolution, which has a background in graph signal processing, learns the features of vertices by heavily relying on the structure of the input graph and therefore makes these methods unsuitable for analysis of unseen data. Creating separate GCN-RS modules and feeding random inputs produces embeddings that are different for each data vector across all sub-graphs. We can then receive additional feature information for every vector in each sub-graph from its neighbors. After averaging the values, we can feed them into a neural network, where the average values are then aggregated. In this way, there is less likelihood of losing information between vertices at the furthest distance. This phenomenon was mathematically proven by Kanatsoulis and Ribeiro (2022).

Furthermore, as with all machine learning procedures, we compared the actual phenotypes of the models with their predicted phenotypes in test partitions. For the GNN-RS, low test MSEs as well as high correlations were observed between actual and predicted values. This supports the fact that GCN models learn better from random feeds (Liu *et al.* 2022). It is also interesting to note that feeding the GCN with complex data structures decreases the model’s capacity to generate meaningful embeddings for learning. There may be information loss during graph creation since the vertices may not be significant enough to be considered nearby.

A limitation of the GCN is its inability to scale if new data needs to be predicted. It is necessary to retrain the entire dataset in order to incorporate new data points. The GraphSAGE model solves this problem (Hamilton *et al.* 2017). However, its injective nature can cause GraphSAGE to have difficulty capturing structural differences in graphs. We attempt to resolve the problem of injectivity by building *N* local sub-graphs that produce different vector embeddings. Following that, we train *N* models based on these sub-graphs. Finally, the final aggregation model uses the trained model weights to produce final predictions. As a result, if a new individual’s phenotype needs to be predicted through GCN-RS, we simply load the aggregated model and feed it the data. Retraining the entire model is thus not necessary.

GraphSAINT is another method that proposes an inductive graph sampling method for efficient training of GCN (Zeng *et al.* 2019). Here, a sampler is used to estimate the probability of sampled nodes and edges. In each batch, an appropriately connected sub-graph is selected. GraphSAINT then trains the model on the sampled sub-graph by building the full GCN on it, and by updating its weights after forward and backward propagation. With GraphSAINT’s sub-graph-based sampling method, nodes are sampled to form sub-graphs, which will improve layer connectivity. In addition, GraphSAINT proposes explicit conditions for eliminating graph sampling bias. When each layer embedding is learned separately, the normalization technique and sampling probability are analyzed.

The GCN-RS method proposed in this paper combines the best features of both the GraphSAGE and GraphSAINT methods by setting a fixed set of neighbors for the creation of the sub-graphs. Further, it is also important to note that the distance between these neighbors is based on the genomic distance between each individual, as well as ensuring that vertices are always connected. There are two advantages to the proposed method, the first being that it has the ability to control the size of the embeddings fed to the network, which allows sub-sampling to train on relevant details as quickly as possible. In addition, because the relationship is based on distance, only individuals who are closely related are included in the sub-graphs, so normalizing the embeddings on the basis of distance is not necessary.

### Conclusion

First, we represent the genomic relationships between individuals in a graph format. Then, we train one global GCN model and one local sub-sampling GCN-RS model that aggregate messages from the individuals nearest neighbors in the graph via neural network embedding and tune the hyper-parameters using out-of-sample validation data. When evaluated on one simulated and three real datasets, the GCN-RS method consistently produce lower test MSE and higher accuracy than the GCN, CNNGWP, and BGBLUP methods. This indicates that the GCN-RS method is a promising method for graph-based ensemble learning. Furthermore, the GCN-RS approach can be scaled up to large datasets without sacrificing performance. This makes it an attractive option for real-world applications. Future research could explore optimizing graph sampling strategies to further enhance the performance of the GCN-RS method. Additionally, investigating the effects of different sampling techniques on various types of graphs could provide valuable insights. Lastly, integrating adaptive sampling mechanisms might lead to even more efficient and accurate graph-based ensemble learning models (Huang *et al.* 2018).

## Supplementary Material

jkae216_Supplementary_Data

## Data Availability

The code and datasets used in the study are available at - https://github.com/ragsGo/GNN/ Supplemental material available at G3 online.
