# Supplementary material for: Sub-sampling graph neural networks for genomic prediction of quantitative phenotypes
Source: G3 (Bethesda). 2024 Sep 9;14(11):jkae216. doi: 10.1093/g3journal/jkae216 (PMC11540326; doi:10.1093/g3journal/jkae216)
Supplement: jkae216_Supplementary_Data [file jkae216_supplementary_data.pdf]

Subsampling graphical neural networks for genomic prediction of quantitative phenotypes: supplementary document

1. FIGURES AND TABLES

A. Layer propagation rule comparison between MLP and GCN

B. Summary of graph representation of the datasets

Dataset	Vertices	Edges	Min Degree	Max degree
QTLMAS	1680	4951	5	14
Wheat	335	1150	5	49
Mice	1016	2962	5	12
Pig	1765	5526	5	43

Table S1. Summary of graph representation of the datasets used (No. vertices, No. edges and the degree at each vertex)

C. Graphic representation of wheat, mice, QTLMAS, and pig datasets

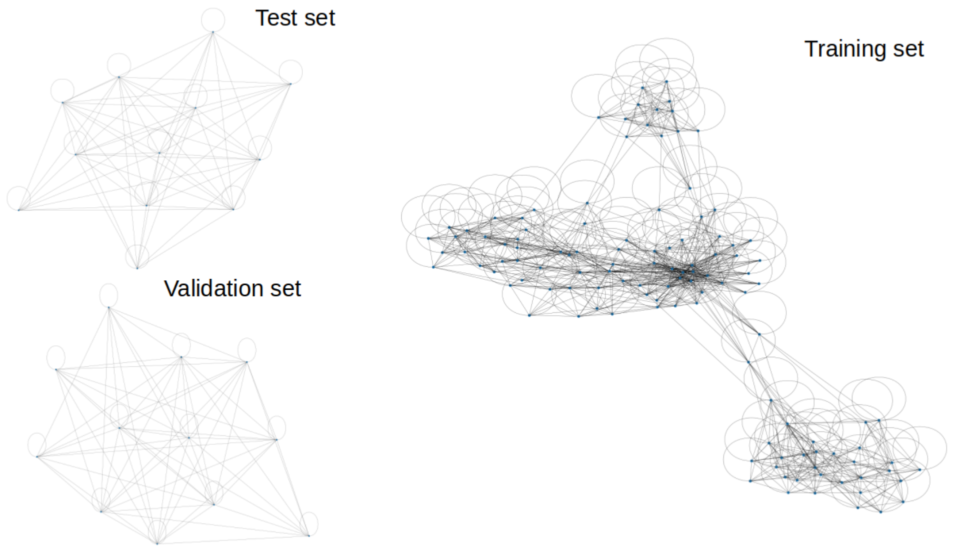

Fig. S1. NN graphs of training, test and validation set split from the wheat dataset

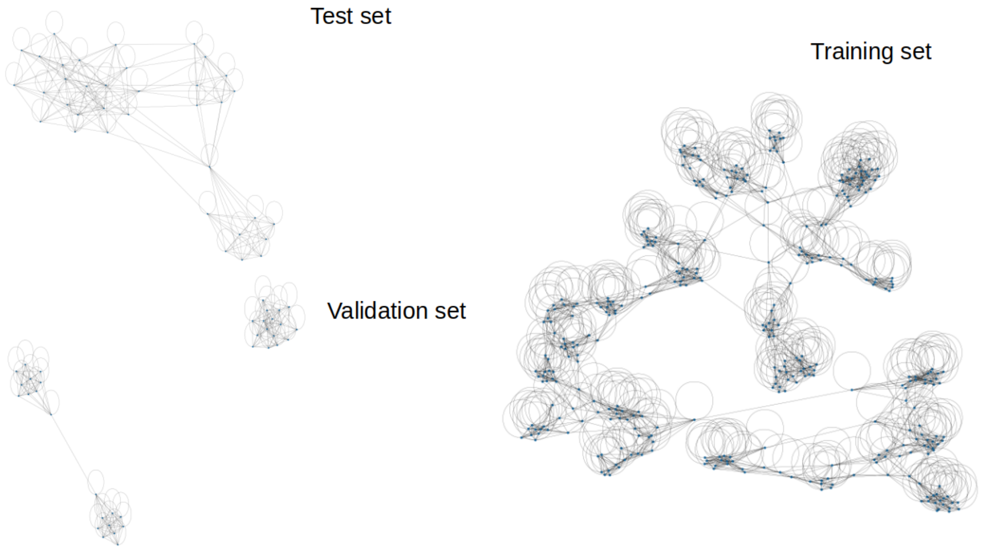

Fig. S2. NN graphs of training, test and validation set split from the mice dataset

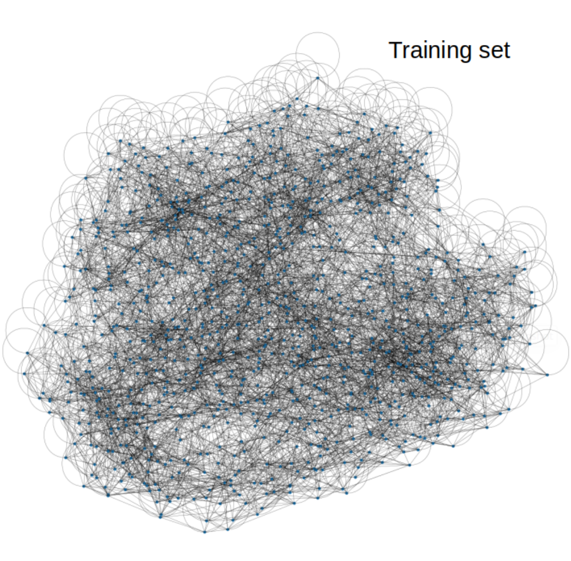

Fig. S3. NN graphs of training, test and validation set split from the QTLMAS dataset

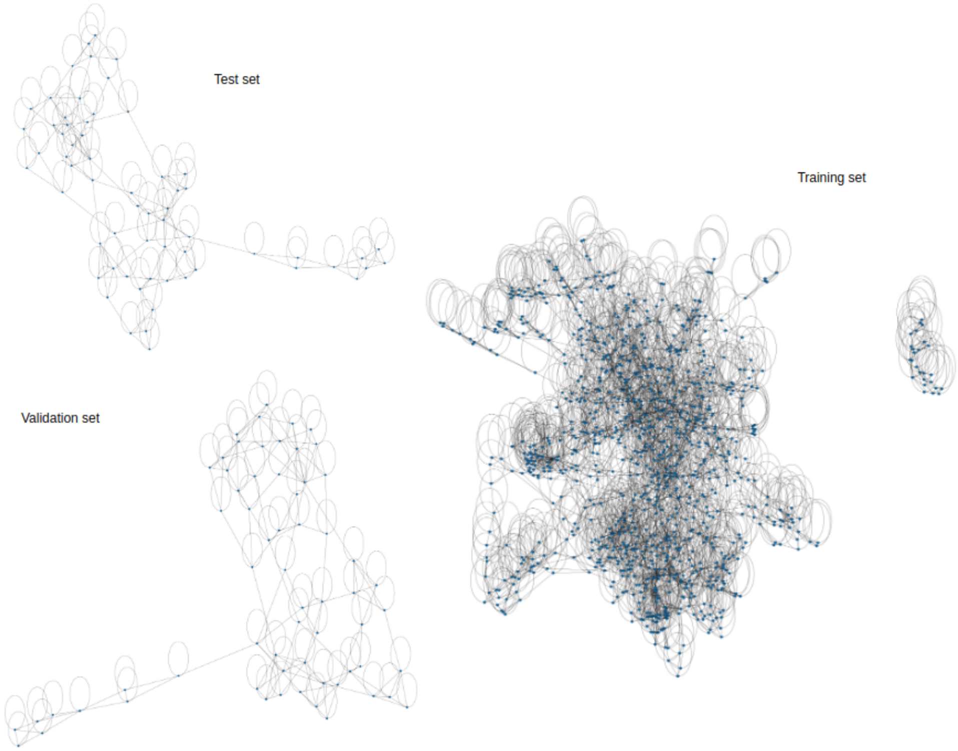

Fig. S4. NN graphs of training, test and validation set split from the pig dataset

D. MSE improvement comparison

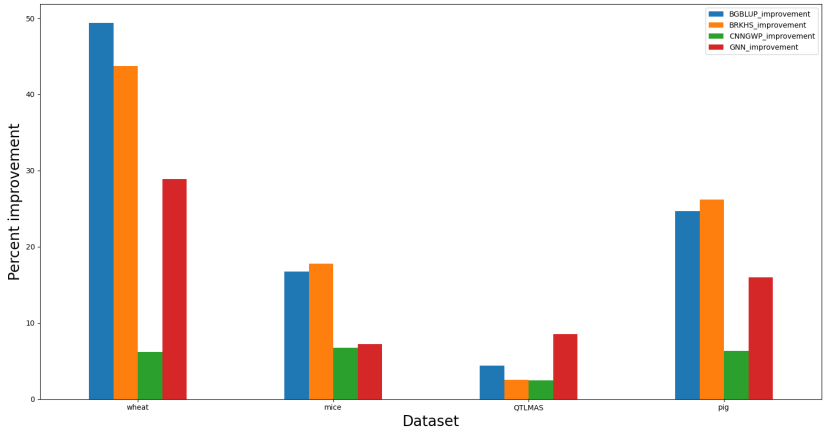

Fig. S5. MSE improvement comparison between GNN-RS and the other datasets

E. GCN training process for the entire graph and subsampling

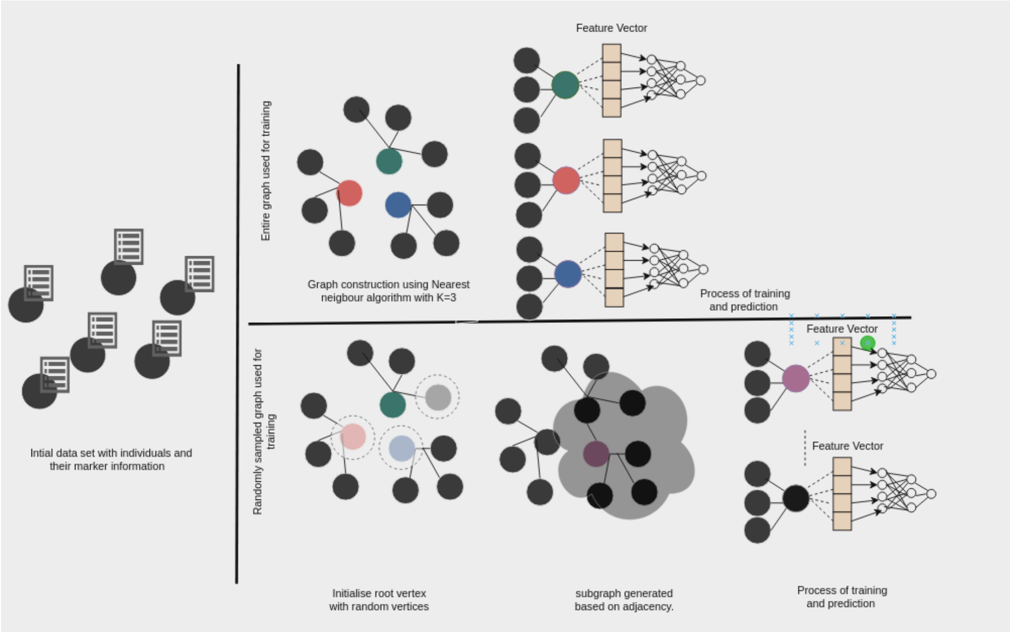

Fig. S6. GCN training process for the entire graph and subsampling
